# Associations of extracurricular physical activity patterns and body composition components in a multi-ethnic population of UK children (the Size and Lung Function in Children study): a multilevel modelling analysis

**DOI:** 10.1186/s12889-019-6883-1

**Published:** 2019-05-20

**Authors:** Lander S. M. M. Bosch, Jonathan C. K. Wells, Sooky Lum, Alice M. Reid

**Affiliations:** 1 Department of Geography, University of Cambridge, Downing Place, Cambridge, CB2 3EN UK; 2 UCL Great Ormond Street Institute of Child Health, London, UK; 3 Respiratory, Critical Care & Anaesthesia section, UCL Great Ormond Street Institute of Child Health, London, UK

**Keywords:** Body composition, Fat mass, Fat-free mass, Extracurricular physical activity, Ethnicity, Childhood overweight and obesity, Epidemiology, Health geography

## Abstract

**Background:**

Body Mass Index (BMI) is a common outcome when assessing associations between childhood overweight and obesity and physical activity patterns. However, the fat and fat-free components of BMI, measured by the Fat Mass Index (FMI) and Fat-Free Mass Index (FFMI), may show contrasting associations with physical activity*,* while ethnic groups may vary in both physical activity patterns and body composition. Body composition must therefore be evaluated when assessing the associations between childhood overweight and obesity and physical activity in multi-ethnic populations.

**Methods:**

This cross-sectional study investigated associations of BMI, FMI and FFMI z-scores with extracurricular physical activity for 2171 London primary schoolchildren (aged 5–11 years) of black, South Asian and white/other ethnicity. Multilevel mixed-effects ordered logistic modelling was used, adjusting for age, sex and family and neighbourhood socioeconomic status as potential confounders.

**Results:**

Controlling for ethnicity and individual, family and neighbourhood socioeconomic confounders, actively commuting children had significantly lower Odds Ratios for being in high BMI (Odds Ratio (OR) = 0.678; 95 % Confidence Interval (CI) = 0.531 − 0.865; *p* − value = 0.002) and FMI z-score groups (OR = 0.679; 95 % CI = 0.499 − 0.922; *p* = 0.013), but not FFMI z-score groups, than passive commuters. Children doing sports less than once a week had lower Odds Ratios for being in high BMI (OR = 0.435; 95 % CI = 0.236 − 0.802; *p* = 0.008) and FFMI (OR = 0.455; 95 % CI = 0.214 − 0.969; *p* = .041) z-score categories compared to daily active children. Differences in FMI between groups did not reach the significance threshold. A trend towards statistical significance was obtained whereby children’s complete inactivity was associated with higher odds for being in higher BMI (OR = 2.222 : 95 % CI = 0.977 − 5.052; *p* = .057) and FMI z-score groups (OR = 2.485 : 95 % CI = 0.961 − 6.429; *p* = .060). FFMI z-scores did not show a similar trend with complete inactivity.

**Conclusions:**

Active commuting was objectively associated with lower adiposity, while more frequent extracurricular sports participation was correlated with greater fat-free mass accretion. These relationships were independent of ethnicity and individual, family or neighbourhood socioeconomic confounding factors.

**Electronic supplementary material:**

The online version of this article (10.1186/s12889-019-6883-1) contains supplementary material, which is available to authorized users.

## Background

Excessive fat accumulation in children is a major risk factor for a plethora of short-term physical and socio-psychological health conditions, as well as for early-onset morbidity and mortality in adulthood [1, 2]. Body Mass Index (BMI) is still the most widely adopted index of children’s fatness, as its simplicity and the low cost of data collection and interpretation are useful when measuring large samples. However, the limitations of using BMI in the study of childhood overweight and obesity are well-established [3], as it does not differentiate fat and fat-free body mass components [4, 5]. Moreover, both BMI itself and the thresholds at which high BMI is associated with ill-health vary with age, sex and ethnicity [6].

The development of age- and sex-adjusted BMI z-scores for children aged 5 to 19 years old resolves the issue of age and sex variability [7]. Conventionally, the cut-off points for overweight and obesity are set at the 85th and 95th percentile boundaries, respectively [8]. However, BMI z-scores systematically underestimate body fatness in South Asian children, while overestimating adiposity in black African children [9, 10]. One way to remedy this is to apply adjustment factors, recently published for these ethnicities in British 4- to 12-year-olds [11]. However, the lack of direct data on body composition hinders elucidation of the underlying, multifactorial drivers of ethnic differences in obesity risk, such as genetic, socioeconomic, cultural and family routine variables [12]. Similarly, the different health implications of fat and fat-free mass make the distinction between them vital in the determination of the risks posed by adiposity, as obesity-related health risks are primarily the consequence of excessive fat accumulation rather than total weight [4, 5].

These differential health implications are particularly relevant when studying the associations of physical activity (PA) with childhood overweight and obesity. PA not only contributes to fat oxidation, but may also stimulate muscle development [13]. Increased PA, and consequently fat-free mass, could therefore entail a higher overall BMI. However, the impact of PA on body composition and BMI and its potential health benefits means that high BMI due to high PA has very different implications compared to high BMI deriving from high fatness. BMI thus needs to be divided into fat and fat-free body mass indices, abbreviated as FMI and FFMI respectively [14, 15].

### Study aim

We aimed to study the association of PA with body composition in a multi-ethnic sample of UK primary schoolchildren. Ethnicity was taken into account with the objective of demonstrating more reliably the association of both components of body composition with the two prime contributors to children’s daily extracurricular PA [16]: competitive or leisurely sports participation outside of school hours, and active commuting to and from school.

## Methods

### Participants

This cross-sectional analysis used data from a sample of London primary schoolchildren who participated in the Size and Lung Function in Children (SLIC) study carried out at the Great Ormond Street Institute of Child Health, University College London, UK [17]. Between December 2010 and June 2013, data on body composition, PA and socioeconomic status were collected for 2171 children aged 5 to 11 years, attending thirteen schools in London. These schools were purposefully sampled based on their diverse geographical location and education performance. Moreover, schools were selected to have strongly diverging ethnic compositions. Together, this ensured the collection of a SLIC sample with a broad spectrum of ethnic and socioeconomic backgrounds [17, 18]. Further detailed information on the recruitment of schools and participants, their representativeness and data collection methods of the SLIC Study can be found elsewhere [17, 18].

### Measures

Anthropometric and body composition data were collected by the SLIC team during school visits. These included the child’s height in centimetres, total body mass in kilograms, and fat-free body mass (FFM) in kilograms estimated from bio-electrical impedance analysis (BIA) using standard instrumentation (Tanita BC418, Tanita Corporation, UK). FFM was calculated from raw BIA data using the multi-ethnic calibration equation of Lee et al. [19], generated from the same population using deuterium dilution as the reference method. Fat mass (FM) in kilograms was calculated by subtracting the FFM from total body mass.

BMI for SLIC children was calculated by dividing total body mass in kilograms by the square of their height in metres. These were converted to age- and sex-specific percentiles using the 2007 World Health Organization BMI reference charts [20]. Analogous to the calculation of BMI, FFM and FM were then divided by the square of height in metres to give fat-free mass index (FFMI) and fat mass index (FMI). The need for age- and sex-adjusted FMI and FFMI reference charts, similar to those for BMI, has been highlighted in prior research, and these have been developed for specific groups of children [21–24]. However, no adequate reference charts currently exist for the age group and ethnic mix of our sample of UK participants. Hence, the raw FMI and FFMI scores were further converted to within-study age- and sex-specific z-scores using multiple regression analysis. Age- and sex-adjusted z-scores and percentiles of FMI and FFMI are thus sample-specific, though they can be considered to be representative for the London-wide population of primary schoolchildren aged 5–11, given the large diversity in terms of age, sex and ethnicity of included participants [17, 18]. To address skew, FM was first natural log-transformed.

Following the guidelines of the Centers for Disease Control and Prevention [8], the 5th, 85th and 95th percentiles were used as class boundaries to assign each individual child in the SLIC database to one of four categories of age- and sex-adjusted BMI (underweight, normal weight, overweight or obese). In analogy, age- and sex-adjusted FMI and FFMI of SLIC children were also subdivided into four categories, which were designed to be equivalent to the categories for BMI (low, normal, moderate or elevated FM and FFM for height, respectively). Hence, for both FMI and FFMI measures, the 5th, 85th and 95th percentiles were selected as class boundaries.

Data on SLIC children’s extracurricular PA were also directly collected via a questionnaire during the school visits for two key components of their extracurricular activity [16]. Firstly, the frequency of participation in a series of twelve sports was gauged. These included the potentially low-cost activities of running/jogging and cycling, regular outdoor activities (football, cricket, skating/rollerblading), regular indoor activities (gymnastics, swimming, badminton/tennis, basketball, dancing, judo/boxing, weightlifting) and an ‘other’ category where less common sports could be indicated. Possible frequencies ranged from ‘never’ through ‘less than weekly’, ‘weekly’ and ‘most days’ to ‘daily’. A composite measure was constructed, providing an overview of the overall sports participation of each child. Second, children were asked for the dominant mode of transport when commuting from home to school. Afterwards, parents and guardians were asked the same question through a parental questionnaire. Responses were classified as ‘active commuting’ if the child predominantly walked or cycled, or ‘passive commuting’ where car, bus or metropolitan railway/underground were the dominant modes of transport. If both active and passive transport constituted significant parts of the commute, this was labelled ‘mixed commuting’. Where parent and child responses differed, this was retained by their inclusion in a ‘disagreement’ category.

Data on sex, age and attended school of participating children were directly collected during the school visits. Parents/guardians were asked to provide detailed information on their child’s ethnicity via a questionnaire [17, 18]. Based on this information, children were categorized into three main ethnic groups: black (African or Caribbean ancestry), South Asian (ancestry from the Indian subcontinent) and white/other (European/other/mixed ancestry). The ‘other’ category is added to the ‘white’ category as over 70% of the 364 children belonging to this group of whom the ethnicity is known are half white, by far the largest proportion of any ethnicity. Socioeconomic data were also gathered via the questionnaire [17]. Family socioeconomic status was derived from an inventory of material possessions, summarized in the Family Affluence Scale [25]. Car ownership (zero to two cars) was accounted for separately, given its decisive role in commuting choices [26–28]. Whether or not the child received free school lunches, a reliable indicator of socioeconomic disadvantage in the UK [29], was included as a final indicator of family socioeconomic status. Using the child’s postcode of residence, neighbourhood socioeconomic status was categorized using the 2010 Index of Multiple Deprivation, combining 38 weighted variables across seven domains of deprivation for small-area units [30]. These contextual data for the SLIC sample are summarized in Additional file 1: Table S1.

### Analyses

Statistical analyses were performed in Stata 14.2 (StataCorp 2015, College Station, Texas, USA). *χ*^2^-tests were performed to analyse binary relationships between ethnicity and body composition measures, PA and potential confounders. When analysing the associations of PA and body composition, the nested data structure, whereby pupils are grouped by school, needed to be accounted for. Individuals attending the same school are likely to be more similar to each other compared to those attending a different school [31, 32]. As non-white children in England are unequally confronted with socioeconomic and obesity risk factors [33], the distribution of and exposure to these risk factors differed by school, according to their ethnic composition. This clustering of more similar subjects in groups by school violates the assumption of independence of observations and generates a need for school-specific information [34]. Compositional effects must be distinguished from contextual ones. This can be done through a multilevel analysis on two levels [35]. The first level includes the PA of the child, as well as her or his individual characteristics (ethnicity, age and sex), and family and neighbourhood socioeconomic status. This captures the specific individual, socioeconomic environment she or he is exposed to. The second level then groups the children by the school they attend. This corrects for their higher likelihood of similarity due to the exposure to more similar environments based on their place of residence and school location. Simultaneously, the mixed-effects regression models designed for the analysis of clustered data must be adapted to fit the ordered categorical nature of the response variables for body composition. Hence, multilevel mixed-effect ordered logistic modelling was performed for BMI, FMI and FFMI on the level of the individual child and the attended school [36]. Results are shown as Odds Ratios (OR) for being in higher BMI, FMI or FFMI categories, with 95% Confidence Intervals. The critical significance level for all models was set at *p* < 0.05. Participants for whom anthropometric, body composition, PA or Index of Multiple Deprivation data were lacking, or who were considered to be unhealthy at the time of data collection, were excluded from analysis.

## Results

### Descriptive statistics

For 1889 SLIC children who were healthy at the time of data collection, data were available for each of the anthropometric, body composition, PA and Index of Multiple Deprivation measures (87.0% of the total sample). Table 1 shows that for 18.7% of children, their age- and sex-adjusted BMI z-score percentile placed them in the obese category, with another 12.4% falling in the overweight category. For FMI, 9.1% had a score between the 85th and 95th percentiles, and another 6.1% scored above the 95th percentile. For FFMI, similar percentages were obtained. Results for self-reported extracurricular PA suggest that 44.1% of children engaged in sports every day, with only 1.4% being completely inactive. Nearly half of the children commuted actively to school. One-third predominantly used passive means of transport. In one out of five cases, however, parent and child commuting reports were inconsistent (21.1% of responses). Due to its size, this disagreement group has been retained as a separate category in the statistical models.

**Table 1:** Body composition and PA measures for the sample of SLIC children in the current study

Variable	Number of SLIC children	Percentage (%)
Age- and sex-adjusted BMI z-score		
< 5th percentile, ‘underweight’	75	4.0
5th–85th percentile, ‘normal weight’	1227	64.9
85th–95th percentile, ‘overweight’	234	12.4
> 95th percentile, ‘obese’	353	18.7
ln(FMI) z-score		
< 5th percentile	56	3.0
5th–85th percentile	1546	81.8
85th–95th percentile	172	9.1
> 95th percentile	115	6.1
FFMI z-score		
< 5th percentile	61	3.2
5th–85th percentile	1564	82.8
85th–95th percentile	167	8.9
> 95th percentile	97	5.1
General Sports Frequency		
Never	26	1.4
Less than weekly	73	3.8
Weekly	476	25.2
Most days	481	25.5
Daily	833	44.1
Dominant mode of commuting		
Active	883	46.7
Passive	598	31.7
Mixed	9	0.5
Disagreement child-parent	399	21.1

*PA* Physical Activity, *BMI* Body Mass Index, *FMI* Fat Mass Index, *FFMI* Fat-Free Mass Index

Further characteristics are summarized in Additional file 1: Table S1. 53.7% of the sample were female. All age groups between 5 and 11 years were well-represented, while black and South Asian children accounted for over half of the total sample. The majority of children lived in families with intermediate Family Affluence Scale scores, with a slight tendency towards higher affluence. Nearly a quarter received free school lunches. About a quarter of families did not own a car, while a similar proportion owned two. Most children lived in relatively deprived neighbourhoods, with 32.2% residing in an area in the most deprived quintile for the Index of Multiple Deprivation.

### Binary statistics relating to ethnicity

Table 2 demonstrates that, using BMI, the proportion of black children with overweight or obesity (42.1%) was significantly higher than that for children of white/other ethnicities (29.9%) and South Asian children (22.5%). This contrast was reduced when FMI was analysed, although inter-ethnic differences remained significant. The proportion of South Asian children with FMI percentile over 95 was higher than in the white/other ethnic group (6.8% versus 4.0%). The largest differences were observed for FFMI percentile scores. 24.1% of children of black ethnicity had an FFMI percentile score above the 85th percentile. For the white/other group, this was 14.2%, for South Asians only 4.1%.

**Table 2:** Body composition and physical activity according to ethnicity

Variable	Ethnicity, n (%)			Chi^2^-test
	Black	South Asian	White/Other	Pearson-χ^2^; *p*-value
Age- and sex-adjusted BMI z-score				
< 5th percentile, ‘underweight’	9 (1.8)	46 (8.9)	20 (2.3)	88.8; <.001***
5th–85th percentile, ‘normal weight’	275 (56.1)	353 (68.5)	599 (67.8)	
85th–95th percentile, ‘overweight’	71 (14.5)	46 (8.9)	117 (13.2)	
> 95th percentile, ‘obese’	135 (27.6)	70 (13.6)	148 (16.7)	
ln(FMI) z-score				
< 5th percentile	23 (4.7)	8 (1.5)	25 (2.8)	39.7: <.001***
5th–85th percentile	359 (73.3)	435 (84.5)	752 (85.1)	
85th–95th percentile	63 (12.8)	37 (7.2)	72 (8.1)	
> 95th percentile	45 (9.2)	35 (6.8)	35 (4.0)	
FFMI z-score				
< 5th percentile	7 (1.4)	46 (8.9)	8 (0.9)	163.6; <.001***
5th–85th percentile	365 (74.5)	448 (87.0)	751 (85.9)	
85th–95th percentile	62 (12.7)	15 (2.9)	90 (10.2)	
> 95th percentile	56 (11.4)	6 (1.2)	35 (4.0)	
General Sports Frequency				
Never	5 (1.0)	4 (0.8)	17 (1.9)	19.5; .013*
Less than weekly	21 (4.3)	21 (4.1)	31 (3.5)	
Weekly	112 (22.9)	118 (22.9)	246 (27.8)	
Most days	126 (25.7)	116 (22.5)	239 (27.0)	
Daily	226 (46.1)	256 (49.7)	351 (39.7)	
Dominant mode of commuting				
Active	204 (41.6)	249 (48.3)	430 (48.6)	10.4; .108
Passive	158 (32.3)	159 (30.9)	281 (31.8)	
Mixed	3 (0.6)	2 (0.4)	4 (0.5)	
Disagreement child-parent	125 (25.5)	105 (20.4)	169 (19.1)	

*BMI* Body Mass Index, *FMI* Fat Mass Index, *FFMI* Fat-Free Mass Index

*: *p* < .05; **: *p* < .01; ***: *p* < .001

Significant associations (*p* < .05) on the first level of the multilevel model are in bold

Ethnic groups differed significantly in their overall frequency of sports participation. 71.8% of black children and 72.2% of South Asian children in the SLIC sample participated in sports more than once a week. For those of white/other ethnicities, this was 66.7%. We found no significant inter-ethnic differences in commuting modes.

Additional file 2: Table S2 and Additional file 3: Table S3 illustrate further significant differences between ethnicities. Families of black children scored lower on the Family Affluence Scale compared to white/other families, with South Asians having intermediate scores. Car ownership was lower for black families than for white/other and South Asian families. The findings for free school lunches showed the opposite pattern. Families of black children tended to live in more deprived neighbourhoods than the other groups. The ethnic composition of the schools also differed significantly.

### Multilevel mixed-effect ordered logistic models linking body mass and composition to PA

The results of the multilevel mixed-effect ordered logistic models linking body composition to sports participation are shown in Table 3. Children of black ethnicity had significantly higher childhood overweight and obesity odds for BMI compared to the white/other group, and similar results were obtained for FMI and FFMI. For South Asians, the opposite findings were obtained for BMI and FFMI. Being younger was associated with lower weight status in terms of BMI. Socioeconomic differences were also evident. If the child received free school lunches, she or he had significantly higher odds for elevated BMI, FMI and FFMI, while a positive relationship between high Family Affluence Score and FFMI was found.

**Table 3:** The relationship between measures of body mass index and body composition and frequency of sports participation

Variable	BMI z-score percentile categories		ln(FMI) z-score percentile categories		FFMI z-score percentile categories	
	Wald Chi-Squared = 77.92; *p* < .001		Wald Chi-Squared = 85.47; *p* < .001		Wald Chi-Squared = 85.47; p < .001	
Level 1	O.R. [95% C.I.]	*p*-value	O.R. [95% C.I.]	*p*-value	O.R. [95% C.I.]	*p*-value
Sports Frequency (reference: daily)						
Never	2.222 [0.977–5.052]	.057	2.485 [0.961–6.429]	.060	1.816 [0.619–5.328]	.277
Less than weekly	**0.435 [0.236–0.802]**	.008**	0.466 [0.210–1.341]	.061	**0.455 [0.214–0.969]**	.041**
Weekly	0.970 [0.750–1.256]	.819	0.967 [0.697–1.341]	.841	0.945 [0.676–1.321]	.741
Most days	0.911 [0.707–1.174]	.471	0.998 [0.728–1.369]	.990	0.832 [0.596–1.162]	.281
Sex (reference: female)						
Male	0.843 [0.687–1.035]	.102	0.975 [0.754–1.261]	.846	1.063 [0.815–1.386]	.654
Age at test	**1.110 [1.041–1.183]**	.001**	1.005 [0.928–1.089]	.903	1.030 [0.947–1.120]	.486
Ethnicity (reference: white/other)						
Black	**1.563 [1.183–2.066]**	.002**	**1.809 [1.268–2.580]**	.001**	**1.612 [1.130–2.300]**	.008**
South Asian	**0.607 [0.452–0.815]**	.001**	1.306 [0.920–1.854]	.136	**0.253 [0.157–0.408]**	<.001***
Family Affluence Scale (reference: low)						
Moderate	1.209 [0.816–1.792]	.344	1.344 [0.818–2.209]	.244	1.491 [0.892–2.492]	.127
High	1.107 [0.692–1.771]	.671	1.728 [0.959–3.112]	.069	**1.940 [1.052–3.574]**	.034*
Free school lunches (reference: no)						
Yes	**1.426 [1.087–1.870]**	.010*	**1.681 [1.211–2.334]**	.002**	**1.582 [1.114–2.247]**	.010*
Cars owned (reference: 0)						
1	0.988 [0.749–1.305]	.934	1.040 [0.737–1.466]	.824	0.987 [0.690–1.411]	.941
2	1.052 [0.731–1.513]	.787	0.872 [0.550–1.381]	.559	0.674 [0.414–1.097]	.113
Multiple Deprivation Index (reference: low)						
Intermediate	1.275 [0.914–1.780]	.153	1.116 [0.735–1.696]	.607	1.318 [0.846–2.053]	.222
High	1.385 [0.979–1.960]	.066	1.384 [0.910–2.103]	.129	1.314 [0.828–2.085]	.246
Level 2: Variance on School Level	0.053 [0.012–0.227]		0.048 [0.008–0.298]		0.112 [0.026–0.479]	

*O.R.* Odds Ratio, *C.I*. Confidence Interval, *BMI* Body Mass Index, *FMI* Fat Mass Index, *FFMI* Fat-Free Mass Index

*: *p* < .05; **: *p* < .01; ***: *p* < .001

Significant associations (*p* < .05) on the first level of the multilevel model are in bold

If only conventional BMI percentiles are taken into account, children who were active less than once a week had significantly lower odds of being overweight or obese compared to children who were active daily. The higher Odds Ratios for completely inactive children to be overweight or obese did not reach the significance threshold for BMI. This picture changes, however, as soon as FMI and FFMI are studied. Using these indexes of fat and fat-free mass, children who were active less than weekly were significantly less likely to belong to high FFMI categories compared to daily active children, whereas the results for FMI did not reach the significance threshold. A trend towards statistical significance was also obtained whereby completely inactive children had significantly higher odds of belonging to the highest FMI categories compared to children who engaged in sports every day. FMI and FFMI trends for other sports frequencies were similar to those for BMI.

Table 4 shows the link between BMI or body composition, and commuting to school. BMI data indicate that active school commuting is associated with lower odds of childhood overweight and obesity compared to passive commuting. A similar finding was also obtained for FMI, but not for FFMI. Observations for mixed commuting and the disagreement category did not reach statistical significance.

**Table 4:** The relationship between measures of body mass index and body composition and mode of commuting

Variable	BMI z-score percentile categories		ln(FMI) z-score percentile categories		FFMI z-score percentile categories	
	Wald Chi-Squared = 82.02; *p* < .001		Wald Chi-Squared = 46.21; p < .001		Wald Chi-Squared = 87.64; p < .001	
Level 1	O.R. [95% C.I.]	*p*-value	O.R. [95% C.I.]	*p*-value	O.R. [95% C.I.]	*p*-value
Commuting mode (reference: passive)						
Mixed	0.471 [0.112–1.979]	.304	0.584 [0.099–3.463]	.554	0.751 [0.138–4.076]	.740
Active	**0.678 [0.531–0.865]**	.002**	**0.679 [0.499–0.922]**	.013*	0.918 [0.665–1.267]	.603
Disagreement	1.028 [0.769–1.375]	.850	0.994 [0.696–1.419]	.972	1.451 [0.998–2.110]	.051
Sex (reference: female)						
Male	0.856 [0.698–1.050]	.136	0.987 [0.763–1.275]	.918	1.085 [0.833–1.414]	.546
Age at test	**1.118 [1.049–1.191]**	.001**	1.012 [0.935–1.095]	.770	1.034 [0.951–1.123]	.436
Ethnicity (reference: white/other)						
Black	**1.459 [1.104–1.927]**	.008**	**1.698 [1.191–2.420]**	.003**	**1.547 [1.084–2.209]**	.016*
South Asian	**0.606 [0.453–0.813]**	.001**	1.302 [0.924–1.837]	.132	**0.248 [0.153–0.401]**	<.001***
Family Affluence Scale (reference: low)						
Moderate	1.093 [0.738–1.618]	.658	1.208 [0.737–1.981]	.453	1.382 [0.827–2.307]	.217
High	1.024 [0.641–1.638]	.920	1.574 [0.875–2.832]	.130	**1.888 [1.026–3.474]**	.041*
Free school lunches (reference: no)						
Yes	**1.412 [1.078–1.851]**	.012*	**1.633 [1.178–2.265]**	.003**	**1.534 [1.081–2.177]**	.017*
Cars owned (reference: 0)						
1	0.961 [0.727–1.269]	.777	1.008 [0.713–1.426]	.964	0.990 [0.691–1.417]	.955
2	0.978 [0.679–1.409]	.904	0.819 [0.515–1.300]	.397	0.664 [0.407–1.082]	.100
Multiple Deprivation Index (reference: low)						
Intermediate	1.265 [0.910–1.760]	.162	1.132 [0.747–1.714]	.559	1.317 [0.849–2.043]	.219
High	**1.407 [1.010–1.961]**	.043*	1.417 [0.949–2.116]	.089	1.332 [0.846–2.097]	.216
Level 2: Variance on School Level	0.045 [0.009–0.210]		0.036 [0.005–0.291]		0.092 [0.019–0.441]	

*O.R.* Odds Ratio, *C.I.* Confidence Interval, *BMI* Body Mass Index, *FMI* Fat Mass Index, *FFMI* Fat-Free Mass Index

*: *p* < .05; **: *p* < .01; ***: *p* < .001

Significant associations (*p* < .05) on the first level of the multilevel model are in bold

Ethnicity and confounders showed the same pattern of association with BMI, FMI and FFMI in the commuting models as in the sports participation models, with the exception that high neighbourhood deprivation was now linked to significantly elevated childhood overweight and obesity measured by BMI.

## Discussion

This study examined the associations of specific physical activity patterns with individual body composition components, accounting for ethnicity and individual anthropometric, family and neighbourhood socioeconomic variables in a multi-ethnic sample of UK children, drawn from the SLIC study.

To our knowledge, it is the first to assess the impact of PA on childhood overweight and obesity for primary schoolchildren by simultaneously relating two potential prime contributors to extracurricular physical activity, mode of commuting to school and frequency of sports participation, to children’s body composition. PA has rarely been disaggregated in the study of its relationship to body composition, and no other study has specifically focused on the relationship between PA patterns and various measures of body composition for a large cohort of primary schoolchildren. Our findings show that active commuting to school is inversely associated with BMI, whereas earlier research has provided inconclusive evidence on its adiposity-countering effects [37, 38]. Interestingly, only when BMI is disentangled into its underlying fat and fat-free components, measured by FMI and FFMI, does it become apparent that the inverse relationship obtained is principally due to lower FMI and not FFMI for active commuters. This is in line with other studies looking specifically at adiposity [39, 40]. These findings are independent of children’s ethnicity, age, gender or socioeconomic status.

Studying body composition also sheds light on the impact of sports participation. Frequent sports participation does not consistently lower a child’s likelihood of being overweight or obese as defined by BMI. A large body of studies prior to ours has generated inconsistent findings on this relationship [41]. Whereas some studies found a BMI-lowering effect of sports throughout childhood and adolescence [42–44], others found no relationship [45, 46]. Again, only when considering FMI and FFMI separately, it is shown that that less-than-weekly sports participation is associated with lower FFMI, whilst there is a trend for completely inactive children to have higher fat masses. The lower FFMI percentile scores for children who are active less than once a week are likely due to reduced muscle development [3, 13]. Despite the more complex data collection process, it is thus indispensable to separately collect information on fat and fat-free mass.

Besides demonstrating the influence of PA components on BMI, FMI and FFMI, this study expands our knowledge of confounding factors that affect body composition. In addition to the standard anthropometric variables age and sex, which were accounted for through the use of z-scores and included as individual confounders, the mediating role played by ethnicity and socioeconomic status is underlined [47, 48]. Our analyses were able to address remaining uncertainties related to these confounders [47] by looking at the effect of ethnicity and socioeconomic status on the same individuals for whom BMI, FMI and FFMI data were available. Considering BMI, overall childhood overweight and obesity levels for the selected SLIC sample were comparable to those for primary schoolchildren at the national level, and slightly below the average for London [49]. However, first, strong inter-ethnic differences were evident. Higher childhood overweight and obesity rates were observed for black children, and lower rates for South Asians, in comparison to the white/other group. Using FMI, levels for children of black ethnicity were still consistently higher than those for any other ethnic group. Whilst not significant, the multilevel models showed that South Asians appeared more likely to be in the highest adiposity category than whites/others. Their lower BMI is hence related to their significantly lower fat-free mass development.

A broad range of family and neighbourhood socioeconomic confounders were then accounted for to further elucidate the ethnicity-body composition relationship, as children of different ethnicities were previously found to be unequally exposed to socioeconomic and environmental obesity risk factors [50–53]. In England, black and South Asian primary schoolchildren are more likely to have obesogenic lifestyles than whites [33]. The binary associations in our research indicate strong socioeconomic disparities between ethnicities. Families of black ethnicity scored significantly lower on the Family Affluence Scale and lived in more deprived neighbourhoods compared to the children in the white/other group, with South Asians having intermediate socioeconomic status. Yet, using multivariate models to correct for the association of ethnicity and family and neighbourhood socioeconomic variables, the significant association of ethnicity with body composition was found to be independent of socioeconomic conditions. Black ethnicity was associated with higher total, fat and fat-free body mass indices, and being of South Asian ethnicity with lower total and fat-free body mass compared to white/other children. This independent association of ethnicity with childhood overweight and obesity is in line with other research in the UK and elsewhere that finds ethnic differences to be only partially explained by socioeconomic characteristics [12, 54].

Our results have several broader implications. First, the finding that different modes of PA are associated with different body composition characteristics has to be considered in future research on the PA-childhood overweight and obesity relationship. The observation that active commuting is linked to lower FMI is especially relevant, given that adiposity has been shown to directly impact the short- and long-term morbidity and mortality of children with overweight and obesity [1, 2]. Interventions should therefore target factors that reduce FMI, as they are more likely to reduce the burden of the childhood overweight and obesity epidemic. Second, we would not have observed all the associations if we had looked only at BMI. Even stronger, healthy muscle mass, rather than excess body fat, might make frequently active children appear as overweight or obese when BMI is used as has been previously suggested [3, 13]. Additionally, BMI is influenced by ethnicity, making the use of accurate anthropometric data and a wide range of socioeconomic characteristics indispensable in the study of childhood overweight and obesity.

Our study also has limitations that need to be acknowledged. Being a cross-sectional study, a causal link between childhood overweight and obesity and PA could not be established. Next, although thoroughly selected and tested, it cannot be guaranteed that all relevant confounders have been included. Moreover, the use of three broad ethnic groups does not allow us to take other ethnicities and their differences at finer scales into account. In addition, both measures of PA rely on subjective self-report, and could not be objectively verified. Whilst no data were collected during the UK school summer holiday period during peak summer months (second half of July to October), seasonality potentially affecting outdoor, non-essential PA could not be corrected for. They also do not capture scheduled or free in-school activity, which may vary between pupils of different schools. Lastly, despite the specific ethnic, socioeconomic and geographically diverse sampling, findings for the SLIC dataset only apply to these primary schoolchildren in London. Caution needs to be exerted when expanding hypotheses beyond the scope of this sample.

## Conclusion

Our study demonstrates how various extracurricular PA components predict body composition in a multi-ethnic population from varying socioeconomic backgrounds and, in so doing, contributes to the development of tailor-made interventions to counter childhood overweight and obesity. Moreover, it does so for children predominantly living in deprived conditions, where overweight and obesity tend to cluster. The findings suggest that future interventions could be particularly promising if they consist of a combination of the promotion of active commuting, lowering FMI, and sports participation, raising FFMI. Disentangling the core variables of the childhood overweight and obesity-PA relationship is indispensable for the development of informed policy measures that could contribute to the reversal of the childhood overweight and obesity epidemic. Notwithstanding their more complex data collection process, fat and fat-free body mass data need to be separately collected, to fully understand observed weight status trends, and anthropometric characteristics and socioeconomic status need to be corrected for. The same applies to PA: it needs to be disaggregated into its different components when its relationship with body composition is studied. The continuous omission of this disentanglement is likely to contribute to the substantial and growing body of literature describing counterintuitive and null-findings.

## Additional files

Additional file 1: **Table S1.** Descriptive statistics for the sample of SLIC children included in the current study. (DOCX 13 kb)

Additional file 2: **Table S2.** Potential confounding variables by ethnicity. Table containing descriptive statistics and results of Chi^2^-tests for potential confounding variables by ethnicity. (DOCX 15 kb)

Additional file 3: **Table S3.** Ethnic composition of SLIC schools. Table containing descriptive statistics and results of a Chi^2^-test for the ethnic composition of SLIC schools. (DOCX 13 kb)

## Abbreviations

BIA: Bio-electrical Impedance Analysis
BMI: Body Mass Index
CI: Confidence Interval
FFM: Fat-Free Mass
FFMI: Fat-Free Mass Index
FM: Fat Mass
FMI: Fat Mass Index
IMD: Index of Multiple Deprivation
OR: Odds Ratio
PA: Physical Activity
SLIC: Size and Lung function In Children
UK: United Kingdom
