# Supplementary material for: Associations of extracurricular physical activity patterns and body composition components in a multi-ethnic population of UK children (the Size and Lung Function in Children study): a multilevel modelling analysis
Source: BMC Public Health. 2019 May 20;19:573. doi: 10.1186/s12889-019-6883-1 (PMC6526612; doi:10.1186/s12889-019-6883-1)
Supplement: Supplementary file 1 — Table S1. Descriptive statistics for the sample of SLIC children included in the current study. (DOCX 13 kb) [file 12889_2019_6883_MOESM1_ESM.docx]

| ***Table S1*** | | |
| --- | --- | --- |
| ***Descriptive statistics for the sample of SLIC children included in the current study*** | | |
| **Variable** | **Number of SLIC children** | **Percentage (%)** |
| **Sex** |  |  |
| *Female* | 1 015 | 53.7 |
| *Male* | 874 | 46.3 |
| **Ethnicity** |  |  |
| *Black* | 490 | 25.9 |
| *South Asian* | 515 | 27.3 |
| *White/other* | 884 | 46.8 |
| **Age at test (years old)** |  |  |
| *5-6* | 464 | 24.6 |
| *7-8* | 720 | 38.1 |
| *9-11* | 705 | 37.3 |
| **Family Affluence Scale** |  |  |
| *Low* | 165 | 8.7 |
| *Intermediate* | 1 208 | 63.9 |
| *High* | 396 | 21.0 |
| *Unknown* | 120 | 6.4 |
| **Free School Lunches** |  |  |
| *Yes* | 462 | 24.5 |
| *No* | 1 268 | 67.1 |
| *Unknown* | 159 | 8.4 |
| **Number of cars in the household** |  |  |
| *None* | 438 | 23.2 |
| *One* | 909 | 48.1 |
| *Two* | 434 | 23.0 |
| *Unknown* | 108 | 5.7 |
| **IMD Quintile** |  |  |
| *Least deprived: 1* | 177 | 9.4 |
| *2* | 372 | 19.7 |
| *3* | 356 | 18.9 |
| *4* | 375 | 19.9 |
| *Most deprived: 5* | 609 | 32.2 |

Table S1: Descriptive statistics for the sample of SLIC children included in the current study

IMD: Index of Multiple Deprivation
