# Supplementary material for: Associations of extracurricular physical activity patterns and body composition components in a multi-ethnic population of UK children (the Size and Lung Function in Children study): a multilevel modelling analysis
Source: BMC Public Health. 2019 May 20;19:573. doi: 10.1186/s12889-019-6883-1 (PMC6526612; doi:10.1186/s12889-019-6883-1)
Supplement: Supplementary file 2 — Table S2. Potential confounding variables by ethnicity. Table containing descriptive statistics and results of Chi2-tests for potential confounding variables by ethnicity. (DOCX 15 kb) [file 12889_2019_6883_MOESM2_ESM.docx]

| ***Table S2: Potential confounding variables by ethnicity*** | | | | |
| --- | --- | --- | --- | --- |
|  | **Ethnicity, n (%)** | | | **Chi^2^-test** |
| ***Variable*** | *Black* | *South Asian* | *White/Other* | *Pearson-χ^2^; p-value* |
| **Sex** |  |  |  | 3.5; .176 |
| *Female* | 281 (57.3) | 270 (52.4) | 464 (52.5) |  |
| *Male* | 209 (42.7) | 245 (47.6) | 420 (47.5) |  |
| **Age at test (years old)** |  |  |  | 2.6; .631 |
| *Five to six* | 117 (23.9) | 132 (25.6) | 215 (24.3) |  |
| *Seven to eight* | 179 (36.5) | 190 (36.9) | 351 (39.7) |  |
| *Nine to eleven* | 194 (39.6) | 193 (37.5) | 318 (36.0) |  |
| **Family Affluence Scale** |  |  |  | **110.4; <.001***** |
| *Low* | 59 (12.0) | 46 (8.9) | 60 (6.8) |  |
| *Intermediate* | 320 (65.3) | 358 (69.5) | 530 (59.9) |  |
| *High* | 49 (10.0) | 98 (19.1) | 249 (28.2) |  |
| *Unknown* | 62 (12.7) | 13 (2.5) | 45 (5.1) |  |
| **Free School Lunches** |  |  |  | **262.6; <.001***** |
| *No* | 194 (39.6) | 426 (82.7) | 648 (73.3) |  |
| *Yes* | 198 (40.4) | 18 (3.5) | 43 (4.9) |  |
| *Unknown* | 98 (20.0) | 71 (13.8) | 193 (21.8) |  |
| **Car Ownership** |  |  |  | **166.5; <.001***** |
| *None* | 178 (36.3) | 102 (19.8) | 158 (17.9) |  |
| *One* | 213 (43.5) | 267 (51.8) | 429 (48.5) |  |
| *Two* | 40 (8.2) | 134 (26.0) | 260 (29.4) |  |
| *Unknown* | 59 (12.0) | 12 (2.3) | 37 (4.2) |  |
| **IMD (Quintile)** |  |  |  | **481.7; <.001***** |
| *Least Deprived: 1* | 10 (2.0) | 1 (0.2) | 166 (18.8) |  |
| *2* | 26 (5.3) | 142 (27.6) | 204 (23.1) |  |
| *3* | 45 (9.2) | 175 (34.0) | 136 (15.4) |  |
| *4* | 123 (25.1) | 105 (20.4) | 147 (16.6) |  |
| *Most Deprived: 5* | 286 (58.4) | 92 (17.9) | 231 (26.1) |  |

Table S2: Potential confounding variables by ethnicity; *: p<.05; **: p<.01; ***: p<.001

IMD: Index of Multiple Deprivation
