# Supplementary material for: Associations of extracurricular physical activity patterns and body composition components in a multi-ethnic population of UK children (the Size and Lung Function in Children study): a multilevel modelling analysis
Source: BMC Public Health. 2019 May 20;19:573. doi: 10.1186/s12889-019-6883-1 (PMC6526612; doi:10.1186/s12889-019-6883-1)
Supplement: Supplementary file 3 — Table S3. Ethnic composition of SLIC schools. Table containing descriptive statistics and results of a Chi2-test for the ethnic composition of SLIC schools. (DOCX 13 kb) [file 12889_2019_6883_MOESM3_ESM.docx]

| ***Table S3: Ethnic composition of SLIC schools*** | | | | |
| --- | --- | --- | --- | --- |
|  | **Ethnicity, n (%)** | | | **Chi^2^-test** |
| ***Variable*** | *Black* | *South Asian* | *White/Other* | *Pearson-χ^2^; p-value* |
| **School ID** |  |  |  | **1,4.10^3^; <.001***** |
| *School 1* | 79 (75.2) | 3 (2.9) | 23 (21.9) |  |
| *School 2* | 47 (74.6) | 5 (7.9) | 11 (17.5) |  |
| *School 3* | 5 (3.8) | 1 (0.8) | 124 (95.4) |  |
| *School 4* | 58 (59.8) | 1 (1.0) | 38 (39.2) |  |
| *School 5* | 21 (7.5) | 199 (71.1) | 60 (21.4) |  |
| *School 6* | 70 (82.4) | 3 (3.5) | 12 (14.1) |  |
| *School 7* | 15 (7.2) | 8 (3.8) | 186 (89.0) |  |
| *School 8* | 43 (28.7) | 27 (18.0) | 80 (53.3) |  |
| *School 9* | 22 (27.2) | 7 (8.6) | 52 (64.2) |  |
| *School 10* | 23 (9.2) | 188 (75.5) | 38 (15.3) |  |
| *School 11* | 25 (13.8) | 46 (25.4) | 110 (60.8) |  |
| *School 12* | 59 (42.4) | 19 (13.7) | 61 (43.9) |  |
| *School 13* | 23 (19.2) | 8 (6.7) | 89 (46.1) |  |

Table S3: Ethnic composition of SLIC schools; *: p<.05; **: p<.01; ***: p<.001
